# Bilateral thalamic infarct involving artery of Percheron: a case report

**DOI:** 10.1097/MS9.0000000000001092

**Published:** 2023-07-26

**Authors:** Himal Bikram Bhattarai, Subid Raj Dahal, Manish Uprety, Madhur Bhattarai, Aseem Bhattarai, Rabindra Oli, Sijuka Devkota, Sanjit Kumar Sah, Suraj Parajuli, Chandra Prakash limbu

**Affiliations:** a P.T. Birtacity Hospital and Research Center, Birtamod, Jhapa; b Tribhuvan University, Institute of Medicine, Maharajgunj; c Metro Kathmandu Hospital, Kathmandu, Nepal

**Keywords:** artery of Percheron, bilateral, Thalamic infarct

## Abstract

**Introduction and importance:**

The thalamus and the midbrain have marked variations and overlapping in their blood supply; one of those variations is the artery of Percheron. Artery of Percheron occlusion is a rare cause of infarction in the bilateral thalamus and midbrain.

**Case presentation:**

In this case, a 60-year-old female with chronic hypertension presented with unconsciousness, motor impairments, and oculomotor disorders.

**Clinical discussion:**

Due to highly variable clinical manifestations and possible negative findings during initial imaging, these conditions are often overlooked, causing delays in therapeutic intervention and leading to bad patient prognosis. Various imaging techniques can be used for diagnosis and treatment should be started early. The treatment aims to promote recanalization as soon as possible and prevent future episodes. The involvement of the midbrain is unfavourable.

**Conclusion:**

Early clinical assessment and neuroimaging are vital for timely diagnosis and early administration of therapeutic measures for better patient prognosis.

## Introduction

Highlights

- Bilateral thalamic infraction due to artery of Percheron infraction is a rare event and cause varying degree of infraction of the thalamus and midbrain.
- The patient presents with vague symptoms of oculomotor disorders, drowsiness, coma, hemiparesis, ataxia, and confusion.
- Involvement of the midbrain has an unfavourable prognosis for the patient.
- Early detection with clinical assessment and neuroimaging is essential for the timely administration of therapeutic measures.

The thalamus is considered to be the great sensory gateway to the cerebral cortex which communicates sensory and motor information, controls voluntary movements, maintains awareness, and is also involved in mood and memory and might also affect electroencephalogram waves^1^. The thalamus and the midbrain receive their blood mainly from the vertebrobasilar artery system and the internal carotid artery system, where there is marked variation and overlapping. One of the variations is the artery of Percheron (AOP), which when occluded, causes bilateral paramedian thalamic and mesencephalic infarctions to occur^2,3^.

**FIGURE 1:**
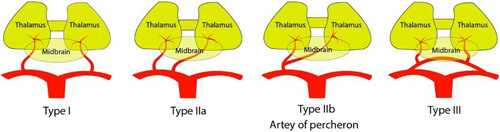
Neurovascular anatomy of thalamus and midbrain showing variation in paramedian artery arising from the P1 segment of posterior cerebral artery.

As described by Percheron, the neurovascular anatomy of the thalamus and midbrain has four normal variations (Fig. 1). The most common is variant I, in which perforating arteries arise from both the left and right posterior cerebral arteries (PCAs). Variant IIa is a rare and asymmetrical variant in which the perforating arteries arise directly from the proximal segment of one of the PCAs. In variant IIb, bilateral perforating thalamic arteries arise from a single arterial trunk known as the AOP, which emerges from the P1 segment of one PCA. This variant supplies the paramedian thalami and the rostral midbrain on both sides. Variant III is an arcade variant with several small perforating branches emerging from a single arterial arch that connects the P1 and P2^4,5^.

AOP is a variation in the vascular supply of the thalamus, found in about one-third of human brains. The AOP infarcts (Type IIb) are rare and are estimated to be in only 0.1–0.3% of all stroke patients^6^. Since the thalamus is responsible for various neurological functions, damage to this area can result in a wide range of clinical manifestations like oculomotor disorders, drowsiness, coma, hemiparesis, ataxia, confusion, and many others depending on the sites affected by the infarct^7^. However, due to its rarity, atypical symptoms and signs, as well as the possibility of it not being visible on initial imaging tests, the diagnosis of AOP infarction is often overlooked^8^. This condition represents an unusual form of ischaemia, making it challenging to identify. When the diagnosis is delayed, the necessary treatment is also delayed, leading to a detrimental effect on the prognosis. This is precisely why we are presenting this case, to highlight the significance of timely diagnosis and treatment in cases of acute AOP infarction and its impact on patient outcomes. This case is in accordance with the CARE reporting checklist^9^.

### Case presentation

A 60-year-old female with a previous history of chronic hypertension was brought to the emergency room by her family after suddenly losing consciousness at home almost one hour prior to presenting at the hospital. Upon arrival, her Glasgow Coma Scale was E1V1M4, and she was noted to have bilaterally dilated pupils that were non-reactive to light. Her pupils were anisocoric, with a right pupil measuring 7 mm and a left pupil measuring 5 mm. The patient was intubated for airway protection and transferred to a neurological unit for further evaluation. Vitals on initial presentation were blood pressure 130/90 mmHg, heart rate 88 beats/min, oxygen saturation 85% in room air with general random blood sugar 158 mg/dl.

**FIGURE 2:**
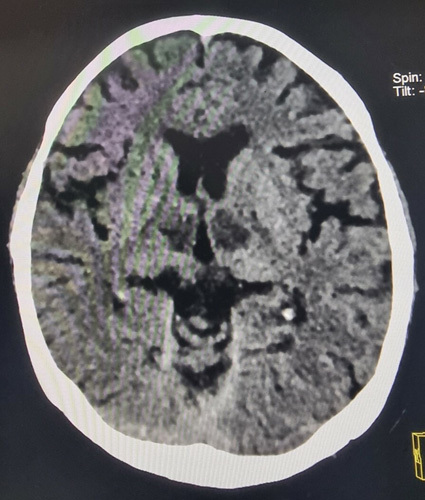
A plain computed tomographicscan of the head showing a hypodense lesion involving bilateral thalami and the rostral midbrain.

During the neurological examination, left-sided hemiplegia and left facial droop were observed. The plantar response was upgoing on the left foot and mute on the right side. Motor power in the left upper and lower limbs was 1 out of 5, while on the right side, it was 2 out of 5 based on Modified Medical Research Council grading. There were no signs of meningeal irritation or neck stiffness. A plain computed tomographic (CT scan) of the head was performed within half an hour of arrival at the hospital, which revealed a hypodense lesion involving bilateral thalami and the rostral midbrain, confirming the diagnosis of an AOP infarction (Fig. 2). The patient’s other systemic examinations were unremarkable, except for decreased air entry bilaterally and occasional wheezing on chest auscultation. She had a history of smoking one pack of cigarettes daily for 20 years and consumed alcohol occasionally.

**FIGURE 3:**
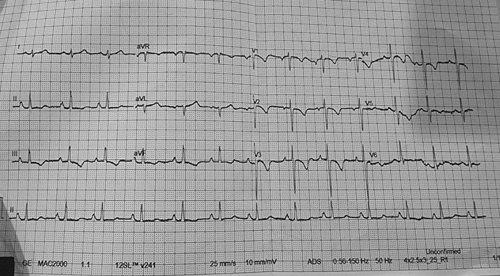
Electrocardiogram showing T wave inversion with a strain pattern.

The laboratory examination revealed slightly elevated haemoglobin and total leucocyte count. The differential leucocyte count, and platelets within normal limits. Prothrombin time was 10 s and the international normalized ratio was (0.9 s). Renal function test, liver function test and lipid profile were normal.(Table 1). The echocardiogram revealed an ejection fraction of 20% with clots in the anterior and apical walls, which were more than 1cm in size, revealing the culprit of the thalamic infarct to be embolic with the source as a left ventricular thrombus. There was T wave inversion with a strain pattern suggestive of ischaemia in electrocardiogram but cardiac enzyme markers (creatinine kinase and troponin I) were negative (Fig. 3).

**Table 1:** Laboratory investigation findings

Laboratory test	Result	Reference range
Haemoglobin	17.6 g/dl	Men: 13.5–17.5 g/dl Women: 12.0–15.5 g/dl
Leucocyte count	11 100 cells/mm^3^	4500–11 000 cells/mm^3^
Neutrophil percentage	60%	40–75%
Lymphocyte percentage	32%	20–45%
Platelets	175 000 cells/mm3	150 000–450 000 cells/mm^3^
Prothrombin time	10 s	9.4–12.5 s
International normalized ratio	0.9	0.8–1.2
Blood urea nitrogen	14 mg/dl	6–20 mg/dl
Creatinine	0.62 mg/dl	0.6–1.2 mg/dl
Random blood sugar level	128 mg/dl	70–125 mg/dl
Sodium	139.9 mEq/l	135–145 mEq/l
Potassium	4.24 mEq/l	3.5–5.0 mEq/l
Erythrocyte sedimentation rate	10 mm/h	Men: 0–15 mm/h Women: 0–20 mm/h
C-reactive protein	8 mg/l	<10 mg/l (low risk)
Total bilirubin	0.9 mg/dl	0.2–1.2 mg/dl
Direct bilirubin	0.23 mg/dl	0–0.3 mg/dl
SGOT (AST)	24.12 U/l	5–34 U/l
SGPT (ALT)	36.21 U/l	7–55 U/l

ALT, alanine transaminase; AST, aspartate transaminase; SGOT, serum glutamate oxaloacetic transaminase; SGPT, serum glutamic pyruvic transaminase.

The patient started antiplatelet therapy, aspirin 75 mg OD, and atorvastatin 10 mg OD. She was then transferred to the intensive care unit for further management. A multidisciplinary team was involved in the patient’s care, including neurologists, neurosurgeons, and critical care specialists. Features of raised ICP (Intracranial Pressure) were suspected from orbital ultrasonogram (USG) revealing thickened optic nerve sheath (7 mm); hence mannitol (20%) 100 ml TDS was initiated. alteplase 9 mg iv bolus stat over 1 min followed by 81 mg continuous IV infusion over 1 h was given. Seizure prophylaxis was started with levetiracetam 500 mg BD. Fluid management was started with normal saline; later, nasogastric tube feeding was started as early as 24 h after admission. Within 1 week of the hospital stay, the patient’s neurological deficits persisted, including left-sided hemiplegia and severe cognitive impairment, with a Mini-Cog Evaluation measured as 0/5. Guardianship was instituted due to the patient’s incapacity, and she was discharged to a skilled nursing facility for extended care.

## Discussion

Compared to unilateral thalamic infarct, which itself is a rare event, the bilateral thalamic infraction is rarer accounting for only 0.6% of all cerebral infarcts and has a worse prognosis due to the neuropsychological and cognitive sequelae that they produce^10^.

Due to its rarity and symptoms that can be confused with those of other neurological conditions, the AOP infarction is difficult to diagnose^6,11^. Bilateral paramedian thalamic lesions can have both vascular and non-vascular differentials, which may have confusing clinical and imaging manifestations (Table 2)^3,4,12^.

**Table 2:** Laboratory investigation findings.

S.N	Vascular cause	
(A)	Top of the basilar artery syndrome	Bilateral thalamic infarcts due to embolic occlusion of arteries in the brainstem, including the PCA, superior cerebellar artery, and pontine arteries.
(B)	Cerebral venous thrombosis	Occlusion of internal cerebral veins, such as the Vein of Galen and Straight Sinus, leads to isolated bilateral thalamic infarcts.
(C)	Chronic hypertensive encephalopathy	Rare cases of chronic hypertension causing bilateral thalamic lacunar infarcts.
S.N	Non-vascular causes	
(A)	Inflammatory conditions	Tuberculosis, malaria, meningoencephalitis, and viral infections can cause bilateral paramedian thalamic lesions.
(B)	Neoplastic conditions	Bilateral thalamic glioma, a rare neoplastic condition, can result in bilateral thalamic expansion and hydrocephalus.
(C)	Demyelinating diseases	Acute demyelinating encephalomyelitis and multiple sclerosis may lead to bilateral thalamic lesions.
(D)	Metabolic disorders	Leigh syndrome, gangliosidoses, Krabbe’s disease, and Wilson’s disease can cause bilateral thalamic lesions.
(E)	Other rare conditions	Creutzfeldt-Jakob disease (spongiform encephalopathy), thiamine deficiency (Wernicke’s encephalopathy), and Fahr’s disease (abnormal calcium deposits) can also be potential causes.

PCA, posterior cerebral artery.

There were found to be four different types of AOP infarction: bilateral paramedian thalamic involvement with rostral midbrain (43%), bilateral paramedian thalamic involvement without midbrain (38%), bilateral paramedian and anterior thalamic involvement with midbrain (14%), and bilateral paramedian and anterior thalamic involvement without midbrain (5%)^11^. Thalamic involvement can be symmetrical (68%) or asymmetrical (32%)^11^.

When acute infarction affects the AOP, it results in the destruction of various combinations of nuclei within the thalami, including the reticular and intralaminar nuclei, associative nuclei, effector nuclei, sensory nuclei, and limbic nuclei. This leads to complex syndromes based on the specific nuclei involved^13^. Common clinical symptoms of bilateral paramedian thalamic infarction include vertical gaze palsy, cognitive impairments (memory deficits, confusion), and consciousness disturbances (coma, decreased vigilance, hypersomnia)^14^. Additional symptoms may manifest, such as disorientation, hemiplegia, cerebellar ataxia, movement disorders, and dysarthria^13^. Vertical gaze palsy marks the involvement of the midbrain tegmentum, the interstitial nucleus of Cajal and the rostral interstitial nucleus of the medial longitudinal fasciculus. Memory dysfunctions are linked to the involvement of the dorsomedial nucleus, located in the paramedian territory supplied by the AOP, which is responsible for memory function. Additionally, impairment of the mammillothalamic tract and anterior nucleus in the anterior territory, usually supplied by the polar artery or AOP, is also associated with memory function. When the anterior thalamic territory is affected, memory impairment tends to be more severe^13^. Oculomotor nerve palsy, including ptosis, ocular movement disorders, and abnormal ocular reflexes, is linked to acute ischaemia of the oculomotor nucleus, which is located near the midline of the mesencephalon. The mesencephalothalamic or thalamopeduncular syndrome, characterized by movement disorders, hemiplegia, and cerebellar ataxia, indicates midbrain involvement^13^. The condition can vary in severity Some individuals may show complete remission, while others may have persistent symptoms like memory loss or mental or oculomotor deficits. There is no distinct pattern of the signs and the clinical presentations are highly variable^14^.

Our patient presented unconscious with non-reactive bilaterally dilated pupils. Left-sided hemiplegia and left facial drop suggested midbrain involvement along with the bilateral thalamus.

Several risk factors have been attributed to AOP infarction and stroke in general, like hypertension, smoking, diabetes mellitus, and dyslipidemia. Etiologies include atherosclerosis, small vessel disease, cardioembolic origin and others^10^. Our patient had chronic hypertension with a 20 pack years history of smoking and when the echocardiogram was done it revealed an ejection fraction of 20% with clots in the anterior and apical walls, which were more than 1cm in size, revealing the culprit of the thalamic infarct to be embolic with the source as a left ventricular thrombus.

The diagnostic process includes clinical assessment and Neuroimaging^11^. The choices for diagnosing AOP infarct early are fluid attenuated inversion recovery (FLAIR) and diffusion-weighted imaging (DWI)^3^. A native head CT is typically the first step in the diagnostic process for suspected posterior circulation ischaemic infarctions in order to rule out haemorrhage because the management of patients with haemorrhagic stroke is very different from that of patients with acute ischaemic stroke^3^. If the first CT scan is negative, additional imaging using brain CT perfusion and CTA is done to look for hidden infarctions. It can be useful to use CT perfusion to distinguish between the parts of the brain that can be saved by interventions and the parts that will infarct regardless of interventions^3^. MRI with T1, T2, FLAIR sequences, and DWI is advised to rule out early-stage infarctions and explore other possible causes if these techniques are unsuccessful in determining the cause^15^. The AOP is too small to be seen on cerebral angiography, so it may not always be appropriate^15^. Infarction of the AOP presents as an abnormal signal intensity on MRI and/or hypoattenuation on CT, involving the bilateral paramedian thalami with or without rostral midbrain involvement^15^. V-shaped hyperintense signal intensity is another notable imaging finding in cases of AOP infarction. Axial FLAIR and DWI images of the midbrain’s pial surface, particularly in the interpeduncular fossa, show this. When the midbrain is involved in cases of AOP infarction, this V sign has been found to be ~67% sensitive. Identification of this distinctive V-shaped hyperintense signal can help with the diagnosis and make it easier to spot Percheron artery infarction.

Due to unavailability of MRI, in our case a plain CT scan of the head was done, which revealed a hypodense lesion involving bilateral thalami and the rostral midbrain, confirming the diagnosis of an AOP infarction.

The treatment objective is to promote recanalization as soon as possible and prevent future episodes. The treatment regimen for AOP occlusion is similar to managing ischaemic strokes in other brain regions, including platelet anti-aggregation therapy, blood pressure and glycemic control and others^16^. As with any ischaemic stroke, the most effective treatment for AOP infarction is thrombolytic therapy, when possible administered within the therapeutic window (4.5 h)^1^. This should be avoided in patients with cerebral haemorrhage found within 3 h or oedema present on CT or MRI. Those who are not suitable for thrombolysis can be given antiplatelet and heparin therapy, Favourable outcome has been found in the study by Zhang *et al*.^13^. Kostanian and Cramer demonstrated a case where selective endovascular thrombolytic therapy for the AOP occlusion showed clinical and imaging improvement after 24 h of onset of symptoms^17^. Thrombolytic therapy via catheterization have demonstrated complete recovery with no gaze palsy or eye deviation^17^. Intravenous heparin 2 days following onset of unconsciousness, dysarthria demonstrated significant cognitive and motor improvement ^18^. Mechanical thrombectomy has been utilized to treat other cerebral arterial occlusions effectively, but usage has not yet been documented for AOP occlusion. Nonemergent cases have been reported to be managed conservatively and were monitored clinically and radiologically following being placed for rehabilitation therapy^18^. Patients should take oral anticoagulants to prevent recurrence^13^

Thalamic infarction generally has a better prognosis than cortical or subcortical lesions, with low mortality and good motor recovery. However, the long-term impact on cognitive function and mood is uncertain^19^. Sequelae such as persistent cognitive dysfunction, memory decline, and intellectual and language dysfunction are seen in most patients^2^. In the case of bilateral paramedian thalamic infarct, 67% of patients achieved functional independence with a Modified Rankin Scale score of less than 2^19^. However, when the midbrain is involved, the percentage of favourable outcomes drops to 25%^19^. This highlights the impact of midbrain involvement on overall prognosis.

Our patient started antiplatelet therapy, aspirin, and atorvastatin. Even with the multidisciplinary team and intense care, the patient had persistent neurological deficits, including left-sided hemiplegia and severe cognitive impairment after 7 days and was discharged to a skilled nursing facility for extended care. The reason for this may be due to a delay in the diagnosis and a narrow therapeutic window that could not be utilized. Since the midbrain was involved, the prognosis was not in the favour of the patient.

Our patient was suspected of raised ICP after orbital USG showed an optic nerve sheath of 7 mm. USG of optic nerve sheath diameter is a noninvasive test that can be performed for suspected raised ICP and has the highest accuracy with an optic nerve sheath diameter of more than 5.8 mm^20^. Raised ICP can result as a complication of stroke, traumatic brain injury, haemorrhage, infection, tumour and other neurological conditions and can result in reduced cerebral perfusion pressure, which can cause cerebral ischaemia or herniation, which may potentially lead to disability and increased rate of mortality^21^.

The diagnosis of AOP infarcts is often delayed due to three main factors: the variety of presenting neurological symptoms, the difficulty of diagnosing AOP infarcts using CT or MRI in the early stages, and the infrequency of AOP infarcts. Consequently, the narrow therapeutic window for thrombolysis cannot be utilized when there is a delay in diagnosis^22^. This highlights the critical importance of early detection to enable the timely initiation of therapy^22^.

## Conclusion

AOP infractions are a rare condition that leads to varying degrees of bilateral paramedian and mesencephalic infraction. Lesions in this region lead to signs and symptoms that are highly variable and difficult to diagnose. This diagnosis and prompt treatment are further delayed in the resource-constrained settings where the patients are more likely to come from remote areas where there is a lack of qualified human resources and modern health facilities, including imaging modalities and therapeutic interventions. Early clinical assessment and neuroimaging is needed for timely diagnosis and early administration of therapeutic measures which favors the patient’s prognosis.

## Ethical approval

None.

## Consent

Written informed consent was obtained from the parents for publication of this case report and accompanying images. A copy of the written consent is available for review by the Editor-in-Chief of this journal on request.

## Sources of funding

None.

## Author contribution

H.B.B., S.R.D., M.U., and M.B. wrote the original manuscript, reviewed, and edited the original manuscript. A.B., R.O., S.D., S.K.S., S.P., and C.P.L. reviewed and edited the original manuscript.

## Conflicts of interest disclosure

The authors declare that they have no conflicts of interest.

## Research registration unique identifying number (UIN)

Name of the registry: None.

Unique Identifying number or registration ID: None.

Hyperlink to your specific registration (must be publicly accessible and will be checked).

## Guarantor

Himal Bikram Bhattarai.

## Data availability statement

All available data are within the manuscript itself.

## Provenance and peer review

Not commissioned, externally peer-reviewed.
